# Dynamic Changes of Platelet and Factors Related Dengue Haemorrhagic Fever: A Retrospective Study in Indonesian

**DOI:** 10.3390/diagnostics12040950

**Published:** 2022-04-11

**Authors:** Imaniar Noor Faridah, Haafizah Dania, Yen-Hsu Chen, Woro Supadmi, Barkah Djaka Purwanto, Mochammad Junaidy Heriyanto, Mahda Adil Aufa, Wei-Chiao Chang, Dyah Aryani Perwitasari

**Affiliations:** 1 Department of Clinical Pharmacy, School of Pharmacy, College of Pharmacy, Taipei Medical University, Taipei 11031, Taiwan; imaniar.faridah@pharm.uad.ac.id; 2 Department of Pharmacology and Clinical Pharmacy, Faculty of Pharmacy, University of Ahmad Dahlan, Yogyakarta 55164, Indonesia; fizadan.djogja@gmail.com (H.D.); wsupadmi@yahoo.com (W.S.); 3 Center for Tropical Medicine and Infectious Disease, Kaohsiung Medical University, Kaohsiung 80708, Taiwan; infchen@gmail.com; 4 College of Medicine, Kaohsiung Medical University, Kaohsiung 80708, Taiwan; 5 Faculty of Medicine, University of Ahmad Dahlan, Yogyakarta 55191, Indonesia; barkah.purwanto@med.uad.ac.id (B.D.P.); m.junaidy@med.uad.ac.id (M.J.H.); 6 PKU Muhammadiyah Bantul Hospital, Bantul, Yogyakarta 55711, Indonesia; 7 PKU Muhammadiyah Kota Yogyakarta Hospital, Yogyakarta 55122, Indonesia; 8 PKU Muhammadiyah Gamping, Sleman, Yogyakarta 55294, Indonesia; adilaufa@gmail.com; 9 Master Program for Clinical Pharmacogenomics and Pharmacoproteomics, School of Pharmacy, Taipei Medical University, Taipei 11031, Taiwan; 10 Integrative Research Center for Critical Care, Wan Fang Hospital, Taipei Medical University, Taipei 11696, Taiwan

**Keywords:** dengue hemorrhagic fever, platelet, risk factor

## Abstract

Dengue is a viral infection caused by the dengue virus (DENV). Dengue infection is a self-limited acute febrile illness caused by four serotypes of DENV (DENV-1~4). Early recognition of high-risk patients would be helpful to reduce mortality rates and prevent severe dengue. Our study aimed to identify factors related to dengue hemorrhagic fever (DHF) based on admission-day data, and further to understand the distribution of biochemical laboratory data in dengue patients. This retrospective study was conducted in hospitals in Yogyakarta city, Indonesia, and involved febrile patients who were admitted to the hospital with a diagnosis of dengue during 2018 and 2020. Logistic regression models were used to identify variables related to DHF. In this study, 1087 patients were included as suspected dengue patients, among them 468 had dengue fever (DF) and 619 had DHF. Over half of the DHF patients were males (55.9%) with an average age of 17.9 years, and with a secondary infection (71.3%). By a multivariate analysis, on-admission laboratory data of thrombocytopenia and hemoglobin showed significant association with DHF. Furthermore, DHF patients had significantly prolonged hospitalizations compared to DF patients. In conclusion, on-admission platelet counts and hemoglobin laboratory data are useful as predictors of DHF especially for suspected dengue patients with the limitations of diagnostic tests.

## 1. Introduction

Dengue is a viral infection caused by the dengue virus (DENV) and transmitted to humans by the *Aedes* mosquito vector. This arboviral disease has spread throughout the world and poses a great public health burden, especially in tropical and subtropical countries [1]. The incidence of dengue reported to the World Health Organization (WHO) has grown significantly over eight-fold in the last two decades from 505,430 cases in 2000 to about 5.2 million in 2019 [2]. Indonesia is one of the dengue endemic countries and has been experiencing a high burden of dengue. Dengue infection has expanded to all regions of Indonesia. All four serotypes of dengue (DENV-1~4) were found around Indonesia [3], with reported dominance of DENV-3 in the Java and Bali regions [4] and DENV-2 in North Kalimantan [5].

The presence of thrombocytopenia is a laboratory feature that is important to monitor in dengue infection. One of the hypotheses related to the mechanism of thrombocytopenia in dengue patients is due to excessive platelet destruction, followed by leakage or hemorrhage during a dengue infection [6]. Thrombocytopenia has been considered as a factor related to the severity of dengue, such as DHF [7] and DSS [8]. Thus, the distributed platelet counts might be used to predict disease progression in dengue patients.

In the current situation of being a country with high endemism, cases of suspected, probable, or confirmed dengue should be provided proper management as early as possible. Laboratory tests should still be used to confirm the clinical diagnosis. Diagnostic tests based on the WHO recommendations include viral isolation and serotype identification, viral nucleic acid detection, and antigen or antibody detection; however, most of these tests are unavailable in every area [9]. Suspected cases of dengue based on the WHO criteria include patients who live in or have travelled to dengue endemic areas, who present with a fever for more than three days, and who present with low white blood cells (WBCs) and/or low platelets with or without a positive tourniquet test. Furthermore, suspected cases confirmed by molecular diagnostic tests could be considered as probable or confirmed dengue [10].

Although the risk of mortality in dengue patients is low [11,12], the pattern of awareness related to dengue infection has changed to other possibilities of severe dengue, such as risks of prolonged hospitalization [12], admission to the intensive care unit (ICU) [13], dengue shock syndrome (DSS) [14], or severe organ involvement [15]. Early recognition of certain factors that contribute to disease severity would be very helpful for clinical providers in managing dengue patients. Thus, the aim in this study was to evaluate factors associated with DHF based on admission-day clinical features and biochemical laboratory data in a population in Yogyakarta city, Indonesia.

## 2. Materials and Methods

### 2.1. Population and Study Design

**Figure 1:**
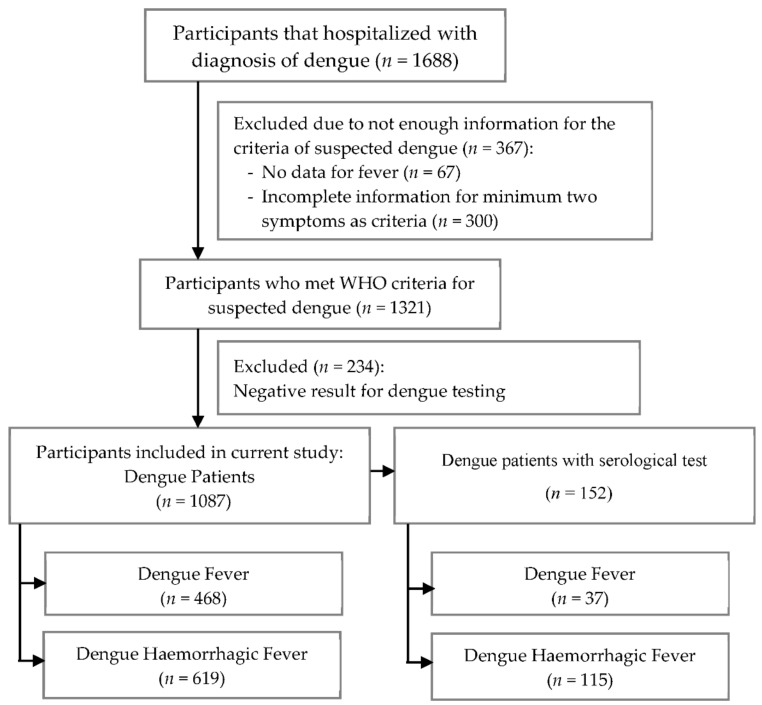
The flow chart for patients’ selection and research design.

This was a retrospective study conducted in hospitals in Yogyakarta, Indonesia. Screening criteria consisted of febrile patients admitted to the hospital with a diagnosis of dengue fever (DF) or DHF between 2018 and 2020. The exclusion criteria were patients who had incomplete information for the criteria of suspected dengue or had negative results from the laboratory for dengue confirmation. The process of patients’ selection, along with the inclusion and exclusion criteria, is described in Figure 1. This study was approved by the ethics committee of the PKU Muhammadiyah Yogyakarta hospital (Ref: 00101/KT.7.4/III/2021) and health research ethics committee of Fakultas Kedokteran dan Ilmu Kesehatan Universitas Muhammadiyah Yogyakarta (UMY) (Ref: 063/EC-KEPK FKIK UMY/XII/2020).

Participants recruited in this study were suspected dengue patients, which were defined as the presence of a fever with additionally a minimum of two or more of the following manifestations: headache, nausea/vomiting, rash, any bleeding manifestation such as gum and gastric bleeding, leucopenia, thrombocytopenia, and an increase in the hematocrit level. Some of the suspected dengue patients (152 patients) had laboratory confirmation tests; rapid tests for immunoglobulin G (IgG), nonstructural protein 1 (NS1) or IgM antibody tests [10,16]. In this study, primary dengue infection was defined as detectable of IgM or NS1 and negative IgG results, while secondary dengue infection was defined by positive IgM or NS1 and IgG results.

Dengue is divided into DF and DHF based on the recommendations from the WHO Regional Office for South East Asia 2011 [17] and the Ministry of Health Republic Indonesia [18]. DF is confirmed by the presence of either a mild febrile syndrome, or other symptoms such as severe headache, muscle/bone pain, nausea/vomiting, and a rash. Moreover, DHF, is characterized by fever, hemorrhagic phenomena, and the presence of thrombocytopenia with hemoconcentration as a sign of plasma leakage [19].

Data were retrospectively collected from patients’ medical records in each hospital. The following information was extracted for each patient: baseline characteristics (sex, age, and length of hospitalization), laboratory data (complete blood cell counts and laboratory confirmation of dengue), and clinical features. Patients’ characteristics and clinical features were recorded on the day of admission, while serological results and laboratory data were checked each day during hospitalization.

### 2.2. Definitions of Serum Biochemical Parameters

Thrombocytopenia refers to a decreasing platelet count of ≤10^5^/mm^3^ [10] and leucopenia is defined as a decreasing number of leucocytes (<5000/mm^3^) [17]. Reference values of laboratory parameters were as follows: hematocrit, 36~40% for children, 40.7~50.3% for adult males, and 36.1~44.3% for adult females; serum glutamic pyruvic transaminase (SGPT) of 7~56 U/L and serum glutamic oxaloacetic transaminase (SGOT) were 5~40 U/L.

### 2.3. Statistical Analysis

DHF-related risk factors including basic characteristics, clinical features, and laboratory data were identified using univariate and multivariate analysis between the DF and DHF groups. Basic characteristics and clinical features were summarized as frequencies and percentages in each group. Descriptive statistics for continuous data are shown as the mean and standard deviation (SD). Analysis of the categorical variables between two groups was performed using ꭓ^2^ test or Fisher exact test. Moreover, a Student’s *t*-test or Mann–Whitney test was used to analyze continuous data. Logistic regression models were used to identify variables related to DHF with and without adjusting age and gender. Significant variables determined by the univariate analysis (*p* < 0.05) were included in the multivariate analysis. The significance level was set to *p* < 0.05. All analyses were performed using SAS software, vers. 9.4 (SAS, Cary, NC, USA) and R software, vers. 4.0.3 (RStudio, 250 Northern Ave, Boston, MA 02210).

## 3. Results

In total, 1688 subjects were admitted to the hospital between 2018 and 2020; however, 1321 participants met WHO criteria as having suspected dengue. Among these participants, 1087 participants were enrolled in a further analysis for which 152 of them were with serological tests (positive results of the IgM rapid test or NS1). Based on the serological tests, 45 patients were primary dengue infection which was defined as detectable of IgM or NS1 and negative IgG results, while 107 patients were secondary dengue infection which was defined by positive IgM or NS1 and IgG results. A flow chart of this study illustrated in Figure 1.

### 3.1. Demographics, Clinical Features, and Laboratory Data

The basic characteristics of patients with DF or DHF are presented in Table 1. According to WHO criteria, 468 (43.05%) patients were classified as having DF, while 619 (56.95%) patients had DHF. Mean ages of the study population were 18.94 years in DF group and 17.92 years in DHF group, which did not significantly differ. The highest percentage of dengue patients was observed in adolescents (12~21 years), followed by young adults (21~45 years) and children (2~12 years). Over half of the patients were male (51.89%) with a higher proportion in the DHF group (55.9%) than in the DF group (46.58%). In terms of length of hospitalization, the mean hospitalization in the DHF group was significantly longer at 4.85 days compared to 4.42 days for the DF group.

Among 1087 patients defined as having suspected dengue, 152 (13.98%) were identified as having a positive result of the IgM rapid test or NS1. The majority of patients, 70.39% from a total of 152 cases with laboratory test, were identified as having secondary dengue, and a higher percentage (76.63%) was confirmed among the DHF patients.

In addition to fever, common features of dengue patients during hospital admission were nausea/vomiting (66.97%), headache (35.79%), and abdominal pain (21.71%) (Table 2). Percentages of nausea/vomiting and abdominal pain were significantly higher in the DHF patients than the DF patients. Fatigue was significantly more likely to be present in the DF group than the DHF group. High hematocrit (50.24%) and thrombocytopenia (66.94%) were also significantly higher in the DHF group. Furthermore, there was no difference in leucopenia between DF and DHF patients.

Table 3 summarizes the laboratory results of dengue patients, and showed that mean values of hematocrit (41.67%) and hemoglobin (14.43 mg/dL) were significantly higher in the DHF patients, and, conversely, the mean of platelet count (85.13 × 10^3^/mm^3^) was significantly lower in the DHF patients compared to DF patients. Among other laboratory data, a lower mean of the WBC count and an elevated mean of serum transaminase levels (SGPT and SGOT) were observed on the admission day of dengue patients, however there was no statistically significant difference between DF and DHF patients.

### 3.2. Factors Associated with DHF

After finding the significance factors associated with DHF from the univariate analysis, 11 variables were further included in the multivariate analysis to identify reliable variables related to DHF. The multivariate analysis was conducted on all dengue patients (*n* = 1087) (Table S1) and on dengue patients with laboratory test (*n* = 152) (Table 4). In this study, we found three variables that were consistently significantly associated with DHF: the length of hospitalization, the hemoglobin count, and the presence of thrombocytopenia on the day of admission. Being of male gender, the presence of fatigue and nausea/vomiting, and a high hematocrit level on the day of admission were shown to be statistically significantly associated with DHF in all dengue patients; however, those variables become insignificant in the dengue patients with laboratory test. Although it was not replicable in dengue patients with laboratory test due to the small sample size, the trend indicated similarities of male gender, nausea/vomiting, and high hematocrit levels as possible risk factors of DHF.

### 3.3. Evaluation of Platelet Profiles Related to DHF

The mean platelet count in DHF patients was significantly lower than that in DF patients, and it was present on day 1, day 3, and the last day of hospitalization of all of the suspected dengue patients. However, on the last day of hospitalization for the patients who had a laboratory test, the mean of platelet count did not significantly differ between DF and DHF patients (Table 5). On day 1, the mean platelet count on DHF patients (85.13 × 10^3^/mm^3^) was below 100 × 10^3^/mm^3^ but not in DF patients (104.73 × 10^3^/mm^3^). Furthermore, the mean platelet count declined on day 3 and had significantly increased by the last day of hospitalization (Table 5).

**Figure 2:**
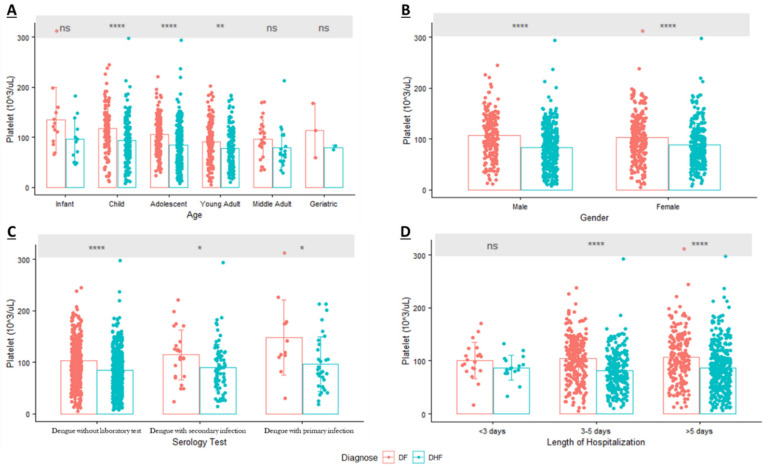
Distribution of Platelet (mean ± SD) in Dengue Patients based on: (**A**). Age; (**B**). Gender; (**C**). Serology Test; (**D**). Length of Hospitalization (ns = Non Significant; * *p*-value < 0.05; ** *p*-value < 0.01; **** *p*-value < 0.0001; DF: Dengue Fever; DHF: Dengue Hemorrhagic Fever).

On the first day of admission, a mean platelet count of <100 × 10^3^/mm^3^ was observed in all age groups of DHF patients; however, it only occurred in young and middle adults among DF patients (Figure 2). Three age groups of children, adolescents, and young adults demonstrated significant differences in platelet counts in DF patients compared to DHF patients. Regarding the distribution of thrombocytopenia in each age group, young adult patients exhibited the lowest mean platelet count in both DF or DHF patients, at about 90.85 × 10^3^/mm^3^ and 77.72 × 10^3^/mm^3^, respectively. Thrombocytopenia was mostly observed on young-adult DF patients (39.91%); however, it mostly occurred in adolescent DHF patients (35.77%).

In terms of gender, results from the multivariate analysis of dengue patients with laboratory test showed that gender was not associated with DHF (Table 4). Consistent with those results, the mean platelet count was lower in DHF patients and significantly differed from that of DF patients; however, it occurred in both male and female patients. In DF, 55.05% of thrombocytopenic patients were female, and conversely, 56.93% of thrombocytopenic DHF patients were male. In another subgroup analysis, the distribution of platelets based on serological tests provided information that patients with a primary dengue infection tended to have a higher mean platelet count than patients with secondary dengue infection (Figure 2). While there was no association between serological tests and DHF due to the small sample size of dengue cases with laboratory test, our results showed that those with a secondary infection tended to have a lower mean platelet count compared to those with a primary infection and tended to develop DHF.

The length of hospitalization was another parameter that showed a statistical difference between DF and DHF patients in the multivariate analysis. After the subgroup analysis, the results were consistent in two groups who were hospitalized three to five days and more than five days. Figure 2 also indicated that patients with thrombocytopenia and who developed DHF tended to have a prolonged hospitalization (>5 days) compared to patients with thrombocytopenia and DF.

## 4. Discussion

Dengue infection remains a serious health problem faced by tropical countries like Indonesia. In terms of resource limitations, awareness of admission-day parameters would be helpful in minimizing the possibility of severe dengue infection and mortality. In this retrospective study, factors were identified to associate with DHF patients, for example, the presence of low platelet counts and high hemoglobin levels on the day of admission. In addition, the length of hospitalization was found to be significantly correlated with DHF.

Demographic data revealed differences between DF and DHF patients of the male gender in this study. Previous reports have indicated the correlation between gender and risk of dengue or severity of dengue [20,21]. It seems that male gender is a risk factor of thrombocytopenia [21] and is also related to the development of severe dengue such as DSS [8]. A prolonged hospital stay was also identified to correlate with DHF. Patients who suffer from dengue infection need more time in hospital, compared to patients with other acute febrile illnesses [22]. Other factors, such as multiple organ dysfunction(s), hypertension, and elevated liver enzymes, can contribute to the longer hospitalization of DHF patients [12].

Dengue infection patients have similar features to other febrile illnesses. Therefore, specific variables need to be identified as predictors of DHF besides validation by laboratory data to confirm dengue. Besides the symptoms of fever, our logistic regression model found that fatigue and nausea/vomiting were related to DHF in all dengue patients; however, results are insignificant in dengue patients with a laboratory test. Despite this finding, the similar trend was observed in both all dengue patients group and the dengue patients with a laboratory test group. Importantly, symptoms of vomiting were also higher in patients with DSS (*p* < 0.001); it occurred in DSS patients rather than in non-DSS patients [8]. Even though this study did not identify significant symptoms as predictors of DHF, some features demonstrated a trend that might associate with the progression to severe disease. Those features are nausea/vomiting and abdominal pain. Thus, patients with such clinical features should be monitored carefully to prevent shock [17].

Other presentations of DHF based on the WHO recommendations are hemorrhagic manifestations such as a positive tourniquet test, petechiae, epistaxis, and gum or gastrointestinal bleeding [17]. The hallmark of DHF, plasma leakage, occurs due to endothelial cell dysfunction caused by many cytokine mediators, mast cell products, or inflammatory lipid mediators [23]. Hemorrhagic manifestations representing abnormalities of hemostasis (blood coagulation) that can be detected by monitoring the prolonged activated partial thromboplastin time (APTT) or thrombin time [24]. In this study, we found that gum bleeding occurred in about 2% of either DF or DHF patients. Gum bleeding is one of the common mild hemorrhagic manifestations that could occur even in non-severe dengue [25].

Thrombocytopenia or a lower platelet count is one of the laboratory parameters that is known to associate to the severity of dengue infection such as DHF [7], DSS [8], and severe organ involvement [15]. The underlying mechanisms of dengue infection –mediated thrombocytopenia are still unclear; however, there are some hypotheses including bone marrow suppression and platelet destruction. Platelet destruction might be caused by DENV nonstructural protein 1 (NS1), a viral protein that is secreted into the blood circulation of dengue patients. NS1 is known to induce immune cell and platelet activation via Toll-like receptor 4 (TLR4), followed by leakage or hemorrhaging during a dengue infection [6]. Furthermore, NS1 antibody titers were significantly higher in DHF secondary infections compared to DF during the critical phase (days 6~7 of fever) [26]. Therefore, monitoring the incidence of thrombocytopenia is important to determine the patients who are most likely to develop severe dengue.

Our study results revealed a significant association between thrombocytopenia (platelets < 100 × 10^3^/mm^3^) and DHF. Platelets in this study demonstrated the same pattern as those of other studies in which a sudden significant drop in platelets in dengue patients occurred between days four and six of fever [27,28]. The platelet counts significantly dropped in DHF patients compared to DF patients on days one and three of hospitalization. Some characteristics such as male gender and secondary dengue patients reported higher percentages of thrombocytopenia on the day of admission and developed DHF. Even though we did not find a significant association between male gender and secondary dengue as risk factors for DHF due to the limited sample size of dengue patients with a serological test, the pattern of these characteristics also reflected the correlation with thrombocytopenia in DHF patients The results were consistent with previous studies [8,20,28].

Another laboratory feature that requires close monitoring is the hematocrit level [17]. Our results showed a strong association between hematocrit and DHF in all dengue cases, however, the significance was not observed in in dengue patients with a serological test diagnosis. Even so, the same pattern of these results showed that a high hematocrit was correlated to DHF (cOR 1.45 in all dengue patients; cOR 1.57 in dengue patients with serological test). A rising hematocrit level is an indicator of plasma leakage that develops in DHF patients, whereas a decreasing hematocrit level is a sign of bleeding. Plasma leakage in dengue infection is caused by extensive immune activation that results in a cytokine storm. There is a hypothesis that in dengue patients with significant plasma leakage, a rising hematocrit level occurs in the febrile phase to the critical phase, and it is associated with decreasing levels of some immune mediators that function in anti-inflammation (IL-1 receptor antagonist (IL-1RA)) and chemotaxis (interferon-γ-inducible protein 10 (IP-10)) [29].

The on-admission laboratory data revealed a relationship between DHF and hemoglobin levels in the multivariate analysis. Hemoglobin levels are known to be significantly correlated with hematocrit in dengue infection [30] In addition to that, hemoglobin and hematocrit levels in dengue patients continued to increase from day 3 of admission until day 10 compared to the non-dengue group (other acute febrile illnesses) [27]. Those results were strengthened by other studies in Surabaya, Indonesia, in which the level of hemoglobin in children and adult patients increased in DHF patients but not in DF patients [31]. A significantly higher level of hemoglobin was also observed in DHF patients during acute febrile illness and on day five of the illness [32]. Both results highlight the importance of recording hemoglobin data in dengue patients as a predictor of DHF and plasma leakage.

There were some limitations in this study. First, this was a retrospective study that used data from available medical records. Therefore, some clinical information was incomplete, such as the virus type, patients’ comorbidities, and day-of-illness (or day-of-fever). Another limitation is the sample size. The number of patients who had laboratory test is small (*n* = 152). In addition, this is a hospital-based study, which is related to severe disease; hence results cannot be generalized to all community-based dengue patients. However, these results should be helpful as supporting data for guiding management in some regions that do not have a well-equipped laboratory or trained staff.

## 5. Conclusions

In conclusion, on-admission platelet counts and hemoglobin laboratory data should be considered as predictors of DHF especially for suspected dengue patients who lack serological or molecular diagnostic tests. Intensive monitoring of these parameters is important to prevent prolonged hospitalization and dengue severity. Future studies with larger samples especially in the second population will be needed to confirm our findings.

## Figures and Tables

**Table 1:** Characteristics of Dengue Patients.

Characteristic	DF (*n* = 468)		DHF (*n* = 619)		Total	*p*-Value	cOR	95%CI	*p*-Value	Adjusted OR	95%CI
	*n*	%	*n*	%							
Age (years), mean ± SD	18.94 ± 13.36		17.92 ± 12.16			0.1894	0.994	0.984–1.003	0.1266	0.993	0.983–1.002
Infant (<2 years old)	13	2.78	12	1.94	25 (2.30)		1			1	
Child (2–12 years old)	140	29.91	196	31.66	336 (30.91)	0.3159	1.517	0.672–3.423	0.3133	1.523	0.672–3.451
Adolescent (12–21 years old)	144	30.77	220	35.54	364 (33.49)	0.2241	1.655	0.735–3.729	0.2404	1.630	0.721–3.688
Young Adult (21–45 years old)	142	30.34	166	26.82	308 (28.33)	0.5705	1.266	0.560–2.864	0.6005	1.245	0.548–2.826
Middle Adult (45–65 years old)	26	5.56	23	3.72	49 (4.51)	0.9311	0.958	0.365–2.514	0.835	0.902	0.342–2.379
Geriatric (>65 years old)	3	0.64	2	0.32	5 (0.46)	0.7441	0.722	0.102–5.095	0.6508	0.636	0.089–4.520
Male Gender	218	46.58	346	55.9	564 (51.89)	**0.0024**	1.453	1.142–1.850	**0.0017**	1.473	1.156–1.877
Length of Hospitalization (days), mean ± SD	4.42 ± 1.35		4.85 ± 1.41			**<0.0001**	1.260	1.150–1.380	**<0.0001**	1.267	1.156–1.388
Laboratory Confirmation Test											
Dengue without laboratory test	431	92.09	504	81.42	935 (86.02)						
Dengue with laboratory test	37	7.91	115	18.58	152 (13.98)						
Primary Infection	12	32.43	33	28.7	45 (29.61)	0.6652	1		0.7782	1	
Secondary Infection	25	67.57	82	71.3	107 (70.39)		1.193	0.537–2.650		1.125	0.496–2.549

DF: Dengue Fever; DHF: Dengue Hemorrhagic Fever; SD: standard deviation.

**Table 2:** Different Clinical Signs and Symptoms of Dengue Patients.

Symptom	DF (*n* = 468)		DHF (*n* = 619)		Total	*p*-Value	cOR	95%CI	*p*-Value	Adjusted OR	95%CI
	*n*	%	*n*	%							
Headache	168	35.9	221	35.7	389 (35.79)	0.9471	0.992	0.772–1.274	0.8255	1.029	0.797–1.329
Rash	11	2.35	13	2.1	24 (2.21)	0.7797	0.891	0.395–2.006	0.9279	0.963	0.425–2.180
Fatigue	28	5.98	21	3.39	49 (4.51)	**0.0442**	0.552	0.309–0.985	**0.0381**	0.54	0.302–0.967
Diarrhea	32	6.84	36	5.82	68 (6.26)	0.4914	0.841	0.514–1.376	0.3638	0.795	0.484–1.305
Gum Bleeding	13	2.78	14	2.26	27 (2.48)	0.5889	0.81	0.377–1.740	0.6416	0.833	0.386–1.799
Nausea/Vomiting	295	63.03	433	69.95	728 (66.97)	**0.0165**	1.365	1.058–1.761	**0.0134**	1.385	1.070–1.792
Abdominal Pain	90	19.23	146	23.59	236 (21.71)	0.085	1.296	0.965–1.742	**0.0452**	1.358	1.007–1.832
Bone Pain	30	6.41	28	4.52	58 (5.34)	0.1726	0.692	0.407–1.175	0.2021	0.706	0.414–1.205
Hemoptysis	15	3.21	35	5.65	50 (4.6)	0.0596	1.809	0.976–3.354	0.0657	1.790	0.963–3.329
Leucopenia (WBC < 5000)	281	73.75	303	73.72	584 (73.74)	0.9922	0.998	0.727–1.370	0.8093	0.961	0.695–1.329
High Hematocrit (HCT > 42)	190	41.13	311	50.24	501 (46.35)	**0.003**	1.446	1.133–1.844	**0.0117**	1.384	1.075–1.782
Thrombocytopenia (PLT < 10^5^)	218	46.78	411	66.94	629 (58.24)	**<0.0001**	2.303	1.798–2.951	**<0.0001**	2.412	1.873–3.106
HCT > 42 & PLT < 50	28	6.01	72	11.73	100 (9.26)	**0.0016**	2.077	1.319–3.271	**0.0028**	2.013	1.273–3.184
Elevated SGPT (SGPT > 56)	39	60.94	40	51.95	79 (56.03)	0.285	0.693	0.354–1.357	0.1835	0.625	0.313–1.249
Elevated SGOT (SGOT > 40)	35	54.69	53	68.83	88 (62.41)	0.0857	1.830	0.919–3.644	0.1545	1.666	0.825–3.364

DF: Dengue Fever; DHF: Dengue Hemorrhagic Fever; WBC: White Blood Cell; HCT: Hematocrit; PLT: Platelet; SGPT: Serum Glutamic Pyruvic Transaminase; SGOT: Serum Glutamic Oxaloacetic Transaminase.

**Table 3:** Biochemical Laboratory Data of Dengue Patients at First Day of Admission.

Variables (Unit), Mean ± SD	*n*	Dengue Fever	*n*	Dengue Hemorrhagic Fever	*p*-Value
Leucocytes (/mm^3^)	381	4720 ± 4370	411	4340 ± 2720	0.435
Hematocrit (%)	462	40.68 ± 6.29	619	41.67 ± 6.04	**0.0027**
Platelet (/mm^3^)	466	104.73 ± 43.92	614	85.13 ± 41.48	**<0.0001**
Blood Glucose (mg/dL)	55	111.60 + 38.12	80	109.84 ± 41.56	0.5863
Hemoglobin (g/dL)	346	14.17 + 2.01	385	14.43 ± 2.03	**0.0487**
SGPT (U/L)	64	83.54 ± 55.85	77	79.49 ± 70.26	0.1893
SGOT (U/L)	64	86.74 ± 62.68	77	121.16 ± 146.66	0.1992
Creatinine (mg/dL)	16	0.80 ± 0.32	36	0.83 ± 0.25	0.5922

SD: standard deviation; SGPT: Serum Glutamic Pyruvic Transaminase; SGOT: Serum Glutamic Oxaloacetic Transaminase.

**Table 4:** Multivariate Analysis to Evaluate Factors Related to Dengue Hemorrhagic Fever in Dengue Patients with a Laboratory Test.

Characteristic	Dengue Patients with Laboratory Test (*n* = 152)		
	*p*-Value	cOR	95%CI
Young Adult (21–45 years old)	0.1449	7.378	0.502–108.349
Male Gender	0.8436	1.113	0.385–3.216
Length of Hospitalization	**0.0018**	1.883	1.264–2.803
Fatigue	0.8846	1.217	0.086–17.250
Gum Bleeding	**0.0115**	0.075	0.01–0.559
Nausea/Vomiting	0.6159	1.338	0.429–4.171
Abdominal Pain	0.6885	1.248	0.423–3.678
High Hematocrit (Hct > 42)	0.4428	1.574	0.494–5.016
Thrombocytopenia (Plt < 100,000)	**0.0029**	6.058	1.848–19.862
Hct > 42 & Plt < 50	0.1746	0.3	0.053–1.707
Hemoglobin	**0.0206**	0.919	0.855–0.987

HCT: Hematocrit; PLT: Platelet.

**Table 5:** Mean of Platelet Count between DF and DHF patients on Day 1, Day 3 and Last Day of Hospitalization.

	Day of Hospitalization	*n*	Platelet Count of Dengue Fever	*n*	Platelet Count of Dengue Hemorrhagic Fever	*p*-Value
Dengue Patients (*n* = 1087)	Day 1	466	104.73 ± 43.92	614	85.13 ± 41.48	**<0.0001**
	Day 3	418	84.97 ± 40.34	564	64.93 ± 34.21	**<0.0001**
	Last Day	409	113.17 ± 43.26	571	103.12 ± 40.50	**<0.0001**
Dengue Patients with Serological Test (*n* = 152)	Day 1	37	125.32 ± 58.78	115	91.28 ± 47.45	**0.0005**
	Day 3	26	91.87 ± 35.36	86	74.57 ± 45.15	**0.0151**
	Last Day	36	111.09 ± 46.83	113	112.74 ± 45.11	0.7478

## Data Availability

All datasets supporting the conclusions in this study are included within the article.

## Supplementary Materials

The following supporting information can be downloaded at: https://www.mdpi.com/article/10.3390/diagnostics12040950/s1, Table S1: Multivariate Analysis to Evaluate Factors Related Dengue Hemorrhagic Fever in All Dengue Patients; Table S2: Distribution of Platelet on Dengue Patients on the First Day of Admission.
