# Supplementary material for: Dynamic Changes of Platelet and Factors Related Dengue Haemorrhagic Fever: A Retrospective Study in Indonesian
Source: Diagnostics (Basel). 2022 Apr 11;12(4):950. doi: 10.3390/diagnostics12040950 (PMC9025030; doi:10.3390/diagnostics12040950)
Supplement: Supplementary file 1 [file diagnostics-12-00950-s001.zip › Supplementary Materials.pdf]

Supplementary Materials:

Table S1. Multivariate Analysis to Evaluate Factors Related Dengue Hemorrhagic Fever in All Dengue Patients

Characteristic	All Dengue Patients (*n* = 1087)		
	p-value	cOR	95%CI
Young Adult (21 - 45 years old)	0.8033	0.893	0.366 - 2.177
Male Gender	**0.0134**	1.395	1.072 - 1.817
Length of Hospitalization	**<.0001**	1.281	1.165 - 1.409
Fatigue	**0.0212**	0.482	0.259 - 0.897
Gum Bleeding	0.0842	0.487	0.215 - 1.102
Nausea/Vomiting	**0.0282**	1.359	1.033 - 1.787
Abdominal Pain	0.172	1.247	0.908 - 1.711
High Hematocrit (Hct > 42)	**0.0128**	1.451	1.083 - 1.945
Thrombocytopenia (Plt < 100000)	**<.0001**	2.307	1.745 - 3.051
Hct >42 & Plt <50	0.6237	1.137	0.68 - 1.900
Hemoglobin	**0.0489**	0.98	0.961 - 1.000

HCT: Hematocrit; PLT: Platelet

Table S2. Distribution of Platelet on Dengue Patients on the First Day of Admission

	Dengue Fever (*n* = 466)			Dengue Haemorrhagic Fever (*n* = 614)		
	Mean ± SD	Thrombocytopenia (*n* = 218)	PLT > 100 (*n* = 248)	Mean ± SD	Thrombocytopenia (*n* = 411)	PLT > 100 (*n* = 203)
Age						
Infant (< 2 years old)	134.07 ± 65.06	4 (1.83)	9 (3.63)	95.81 ± 43.55	8 (1.95)	4 (1.97)
Child (2 - 12 years old)	116.76 ± 43.90	45 (20.64)	95 (38.31)	92.95 ± 42.89	120 (29.20)	76 (37.44)
Adolescent (12 - 21 years old)	105.30 ± 38.06	66 (30.28)	77 (31.05)	83.72 ± 42.31	147 (35.77)	70 (34.48)
Young Adult (21 - 45 years old)	90.85 ± 43.88	87 (39.91)	54 (21.77)	77.72 ± 37.56	119 (28.95)	46 (22.66)
Middle Adult (45 - 65 years old)	96.38 ± 38.01	15 (6.88)	11 (4.44)	79.43 ± 39.60	15 (3.65)	7 (3.45)
Geriatry (> 65 years old)	113.33 ± 54.50	1 (0.46)	2 (0.81)	79.00 ± 5.65	2 (0.49)	0
Gender						
Male	106.20 ± 42.93	98 (44.95)	119 (47.98)	82.70 ± 40.71	234 (56.93)	110 (54.19)
Female	103.45 ± 44.80	120 (55.05)	129 (52.02)	88.20 ± 42.30	177 (43.07)	93 (45.81)
Serological Test						
Dengue without lab test	102.95 ± 42.01	208 (95.41)	221 (89.11)	83.70 ± 39.89	338 (82.24)	161 (79.31)
Dengue with Primary Infection	148.08 ± 72.90	2 (0.92)	10 (4.03)	95.87 ± 53.28	20 (4.87)	13 (6.40)
Dengue with Secondary Infection	114.39 ± 48.61	8 (3.67)	17 (6.85)	89.42 ± 45.11	53 (12.90)	29 (14.29)
Length of Hospitalization						
< 3 days	100.90 ± 34.30	10 (4.59)	10 (4.03)	87.05 ± 23.28	12 (2.92)	5 (2.46)
3 - 5 days	103.82 ± 42.27	117 (53.67)	133 (53.63)	82.07 ± 37.69	170 (41.36)	74 (36.45)
> 5 days	106.28 ± 46.87	91 (41.74)	105 (42.34)	87.14 ± 44.49	229 (55.72)	124 (61.08)

SD: standard deviation ; PLT: Platelet
